# CT Findings and Histological Evaluation of Red Foxes (*Vulpes vulpes*) with Chronic Head Trauma Injury: A Retrospective Study

**DOI:** 10.3390/ani11041010

**Published:** 2021-04-03

**Authors:** Luca Lacitignola, Rossella Samarelli, Nicola Zizzo, Elena Circella, Claudia Acquafredda, Marzia Stabile, Roberto Lombardi, Francesco Staffieri, Antonio Camarda

**Affiliations:** 1 Dipartimento Dell’Emergenze e Trapianti di Organo, Sez, Cliniche Veterinarie e P.A., Università Degli Studi di Bari, Strada Provinciale Per Casamassima Km.3, 70010 Valenzano, Italy; 2 Dipartimento di Medicina Veterinaria, Sez, Patologia Aviare, Università Degli Studi di Bari, Strada Provinciale Per Casamassima Km.3, 70010 Valenzano, Italy; rossella.samarelli@uniba.it (R.S.); elena.circella@uniba.it (E.C.); roberto.lombardi.vet@gmail.com (R.L.); antonio.camarda@uniba.it (A.C.); 3 Dipartimento di Medicina Veterinaria, Sez, Anatomia Patologica, Università Degli Studi di Bari, Strada Provinciale Per Casamassima Km.3, 70010 Valenzano, Italy; nicola.zizzo@uniba.it; 4 Osservatorio Faunistico Regionale Della Puglia, Via Generale Palmiotti, 70020 Bitetto, Italy; 5 Dottorato di Ricerca in “Trapianti di Tessuti ed Organi e Terapie Cellulari”, Dipartimento Dell’emergenza e dei Trapianti di Organi (DETO), Università Degli Studi di Bari, “Aldo Moro”, 70100 Bari, Italy; claudia.acquafredda@uniba.it (C.A.); marzia.stabile@uniba.it (M.S.)

**Keywords:** head trauma, hydrocephalus, chronic brain injury, neurologic disorders, brain atrophy, survival rate, CT, red foxes, road traffic accident, wild carnivores

## Abstract

**Simple Summary:**

Large numbers of wild animals are injured every year in road traffic accidents (RTA) and need first aid and assistance procedures, depending on the severity of the trauma and lesions. Head trauma may lead to traumatic brain injury (TBI), which causes neurologic and behavioral disorders. In this study, we retrospectively evaluated the clinical records of red foxes rescued and clinically evaluated to characterize computed tomography (CT) findings in a cohort of foxes with chronic head trauma and identify survival rates. On average, CT scans were performed 260 days after fox rescue. We speculate that persisting clinical signs could be attributed to TBI. CT scan helped diagnose chronic lesion and their effect on prognostic judgment for animals that should be released to wildlife environments.

**Abstract:**

Large numbers of wild animals are injured every year in road traffic accidents. Scant data are available for rescued wild carnivores, in particular for red foxes. Cases of foxes with head trauma were retrospectively considered for inclusion in this study. Clinical examination, modified Glasgow coma scale (MGCS), computed tomography (CT) examination, therapy, outcome, and post mortem findings of the brain were investigated. In all foxes, cranial vaults lesion occurred in single (67%) or multiple sites (33%). Midline shift and hydrocephalus were observed in this population. The mean survival was 290 (±176) days. In our study, we performed CT scans on average 260 days after fox rescue, and we speculate that persisting clinical signs could be attributed to TBI. In our study, only two foxes were alive at the time of writing. Other foxes were euthanized due to the severity of the clinical signs. CT scans help diagnose chronic lesions and their effect on prognostic judgment for animals released to wildlife environments.

## 1. Introduction

The red fox *(Vulpes vulpes)* is a globally widespread species characterized as a non-migrant with high territorial mobility. Foxes show high adaptability to different habitats and can be particularly common in urban areas due to increasing urbanization and habitat loss. The population is not static because of the foraging and territorial activities and the attempt to find a mate for reproductive purposes [1]. This has led to the colonization of urban and semi-urban areas, with consequent increasing numbers of casualties involving these animals [1,2].

Many wild animals are injured every year in road traffic accidents (RTAs) [3,4] and need first aid and assistance procedures depending on the severity of the trauma and lesions. In domestic carnivores involved in RTA and presented to the veterinary clinics, the diagnosis and further procedures are set up with the support of clinical data and with diagnostic imaging, such as X-rays, computed tomography (CT) scans, and magnetic resonance imaging (MRI). Most of these animals are diagnosed with fractures and other types of injuries, such as head trauma, which may lead to traumatic brain injury (TBI), causing neurologic and behavioral disorders [5]. In domestic carnivores with severe blunt trauma, head trauma occurs in approximately 25% [6], but no data are available for rescued wild carnivores, particularly for red foxes.

In this study, we retrospectively evaluated the clinical records of red foxes rescued from Osservatorio Faunistico Regionale della Puglia (OFR), the Apulian Regional Wildlife Rescue Center, and were clinically evaluated at the Veterinary Clinics and Animal Production Section of the Department of Emergencies and Organ transplantation of the University of Bari. We aimed to characterize CT findings and histological evaluation in a cohort of foxes with chronic head trauma.

## 2. Materials and Methods

### 2.1. Population

Clinical files from OFR for 2017–2019 were retrospectively examined. Admitted cases of red foxes (*Vulpes vulpes*) were selected based on the following inclusion criteria: cases of head trauma without any other musculoskeletal trauma, metabolic disorders, infectious diseases, or dead in 24 h after admission. Files should include sex, presumptive age (juvenile or adult, established based on complete or not growth plate cartilages at X-rays examination), clinical examination, modified Glasgow coma scale (MGCS), CT examination, therapy, and outcome.

Additionally, we evaluated gross anatomy findings of the brain after necropsy and the histological evaluation for definitive diagnosis. Incomplete files were excluded from the analysis.

### 2.2. Clinical Examination

Clinical examinations were performed at the admission of rescued animals, considering signs of wounds (facial, skull), mental status (unremarkable, depressed, delirium, stupor or semi-comatose, comatose), presence of seizures, and other trauma. The MGCS score was calculated by scoring motor activity, brainstem reflexes, and the level of consciousness on a scale of 1 to 6, where a score of 6 represents unremarkable findings, and a score of 1 represents severe impairment, for a total possible score ranging from 3 (least likely to survive) to 18.

### 2.3. CT Examination

CT examinations were performed at Sezione di Cliniche Veterinarie e produzioni animali of the Dipartimento dell’Emergenze e Trapianti di Organo (Bari, Italy). CT was performed under general anesthesia.

All foxes underwent an anesthetic protocol that included an intramuscular premedication with 5 µg/kg of dexmedetomidine (Dexdomitor^®^ 0.5 mg/mL; Orion Pharma, Milan, Italy), 5 mg/kg of ketamine (Ketavet 100^®^ 100, mg/mL; MSD, Roma, Italy) and 0.3 mg/kg of midazolam (midazolam Ibi^®^ 5 mg/mL; Ibi, Aprilia, Italy). Top-ups when needed and time of sedation were recorded. When the sedation was estimated to be adequate for animals’ safe handling, an intravenous (IV) catheter was placed, and fluids (Ringer’s lactate solution) were administered. Propofol (Proposure^®^ 10 mg/mL, Boehringer Ingelheim Animal Health Italia SpA, Milan, Italy) at a dose of 5 mg/kg was used as induction agent. None of the animals were intubated, but all the equipment for intubation and inhaled anesthesia was available if needed; the anesthetic plan was maintained by administering boluses of propofol to effect when needed. Soon after, monitoring was implemented. A multiparametric monitor (B3 VET Multiparametric Monitor; GIMA, Gessate, Italy) was used to record vital parameters, such as heart rate, pulse rate, ECG, SpO2. The respiratory rate, temperature, and color of the mucous membranes were recorded manually. CT scans were performed, on average, 260.8 (±169.9) days after admission.

The CT scanner employed was a 2-slices scanner (Prospeed dual, GE, Chicago, IL, USA). Transverse scans were acquired with a thickness of 2 mm, KV 120, and 200 mAs; reconstruction kernels were for soft, standard, and bone reconstruction, and window width (WW, 100), window level (WL, 50) for the brain, WW (350) and WL (40) for soft tissue, and WW (1500) WL (300) for bone. The acquisition field of view was 18 cm. Scans were performed without and with contrast medium (CM) administration (iopamidol 370 mg/mL, Iopamiro 370, Bracco, Milan, Italy) at a dose of 600 mg/kg IV manually administered. Scans were acquired 7–10 s after CM administration.

CT-considered findings included cranial vault fractures (single or multiple), abnormalities of the parenchyma, considered as hypodense areas in the brain, cerebral hemisphere, or cerebellum as a single lesion, multiple lesions, or a unilateral area in the brainstem. Intracranial hemorrhage was also evaluated as hyperdensity located intra-axially (within brain parenchyma) or extra-axially in the subarachnoid, subdural and epidural spaces. Other reported findings included midline shift, lateral ventricle asymmetry and hydrocephalus. All findings were numbered and scored as 1 = mild—slightly visible, 2 = moderate—modestly appreciable, and 3 = severe—clearly visible.

### 2.4. Therapy

At the arrival of the OFR, all the foxes underwent fluid therapy at shock dose for the first hours. Ringer lactate solution was administered at 50 mL/kg for the first hour, then 20 mL/kg/h was given until stabilization. Once stabilized, the maintenance fluid rate was 2 mL/kg/h.

Due to the risk of cerebral edema, mannitol was intravenously administered at the dose of 1 g/kg over 20 min, and repeated 1 or 2 times after 4 or 8 h as necessary, according to the re-evaluation of the neurological status.

For the subjects presenting seizures, rectal diazepam was given at 0.5 mg/kg as the seizures occurred.

To manage inflammatory status and pain, carprofen was administered subcutaneously at 4 mg/kg once daily until improvement of the pain conditions and enrofloxacin at 5 mg/kg subcutaneously twice daily for the prevention of the infections that could have arisen due to the injuries.

### 2.5. Outcome

The outcome was considered as released animals or alive, but not released, dead, or euthanized. The decision for euthanasia was considered on the basis of the fox’s quality of life-related to release or captivity. Euthanasia was performed under general anesthesia with an overdose of KCl (according to American Veterinary Medicine Association Guidelines for the euthanasia of animals). Survival rates were considered in days from admission to the final outcome. Survived or released animals were considered at the time of writing.

### 2.6. Gross Anatomy and Histological Findings

**Figure 1:**
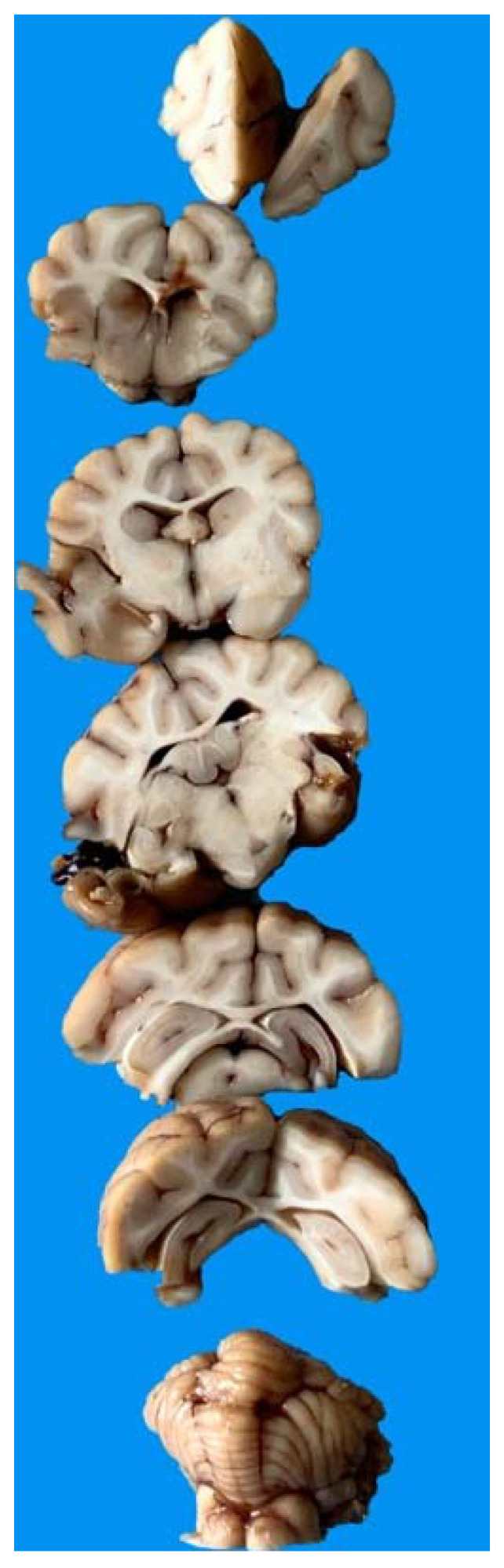
Representative image of a harvested red fox’s brain necropsied and cut through the coronal plane from the frontal (**top**) to the cerebellum (**bottom**).

The necropsy included gross evaluation of the main organs (heart, lung, liver, kidney, and spleen) with particular attention to the brain and further histopathological examination. All samples were immediately stored in 10% buffered formalin. The histological examination was performed for each animal at least 72 h after fixation. The brain was coronally sectioned into 7 serial portions at the level of the frontal lobe, occipital lobe, basal nuclei, thalamus, hippocampus, brain stem, cerebellum (Figure 1).

The tissues of all organs were incorporated into paraffin; the sections were cut into 5 µm thick serial sections and colored with hematoxylin and eosin (H&E) with periodic acid-Schiff (PAS) and examined with D 4000 Leica DMLS microscope equipped with a digital camera (Leica DMC5400, Milan, Italy). The sections were evaluated at 100× and 400× magnification.

### 2.7. Statistical Analysis

Minitab^®^ 19 Statistical Software (Suturentry, UK) was used for statistical examination. Data are summarized as mean ± standard deviation (SD) and inter-quartile range (IQR).

## 3. Results

### 3.1. Population

We evaluated 77 files of red foxes. Of these, only six cases matched the inclusion criteria. Rescued foxes were all males, presumably four young and two adults, with a mean weight of 3.8 ± 0.95 kg (IQR 3.3–4.65).

### 3.2. Clinical Findings

At admission to OFR, four out of six foxes presented mild soft tissue trauma in the ocular region. Table 1 describes the mental status of the investigated foxes at admission. Other clinical signs were nystagmus in one fox, head tilt in a different one and seizure in four cases. The MGCS was 8.3 (±3.0) at admission.

### 3.3. CT Findings

CT scan was performed on average 260 ± 170 days (IQR 75.8–402.5) after admission at the OFR. Table 2 shows the prevalence and related severity of the findings in the studied population of red foxes.

**Figure 2:**
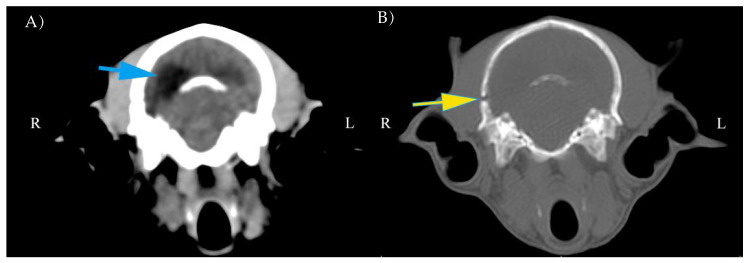
Representative computed tomography (CT) scans at the same position in a red fox with soft tissue reconstruction kernel and window width (WW) (100) window level (WL) (50) for the brain (**A**). Bone reconstruction kernel and WW (1500) WL (300) for bone tissue (**B**). Note the asymmetric left ventricle (blue arrow) and fracture of the cranial vault (yellow arrow) not completely repaired.

**Figure 3:**
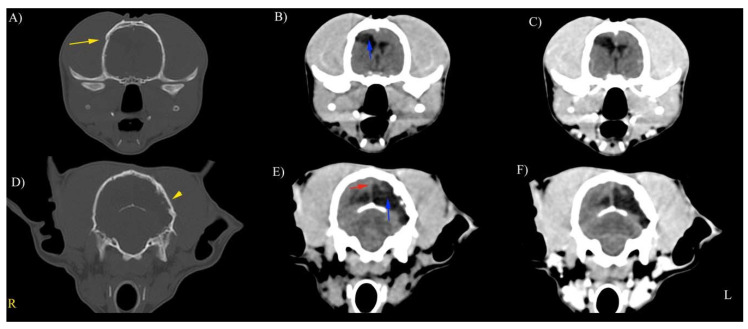
Representative precontrast (**A**–**E**) and postcontrast (**C**,**F**) CT scans in a red fox with soft tissue reconstruction kernel and WW (100) WL (50) for the brain (**A**). Bone reconstruction kernel and WW (1500) WL (300) for bone tissue. Site of fracture (yellow arrow). Ventricles were asymmetrically distended (blue arrow); mild midline shift (red arrow). **Right** (**R**) on the left of the image, **left** (**L**) on the right of the image.

**Figure 4:**
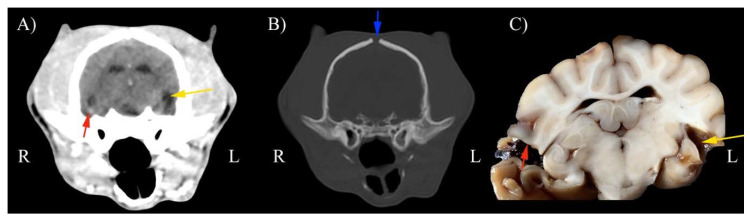
Representative post-contrast CT scans of the same position in a red fox with soft tissue reconstruction kernel and WW (100) WL (50) for the brain (**A**). Bone reconstruction kernel and WW (1500) WL (300) for bone tissue (**B**). (**C**) Gross anatomic section. Note severe hypodense area and related atrophic cerebral area at the level of proliferative left temporal fracture repair (yellow arrows). Hyperdense area (red arrows) corresponding to chronic hemorrhage. Blue arrow represents persistent fontanelle. **Right** (**R**) on the left of the image, **left** (**L**) on the right of the image.

In all foxes, cranial vault lesions occurred in single (67%) or multiple sites (33%). The presence of abnormal parenchyma was constant in all cases, mainly consisting of hypodense areas in the cerebral parenchyma and both intra and extra-axial hemorrhages. Midline shift and hydrocephalus were observed in this population (Figure 2, Figure 3 and Figure 4).

### 3.4. Outcome and Survival

The outcomes included four euthanized foxes, one fox was released, and one is still alive but not released. The mean survival was 290 (±176) days.

### 3.5. Gross Anatomy and Histological Findings

**Figure 5:**
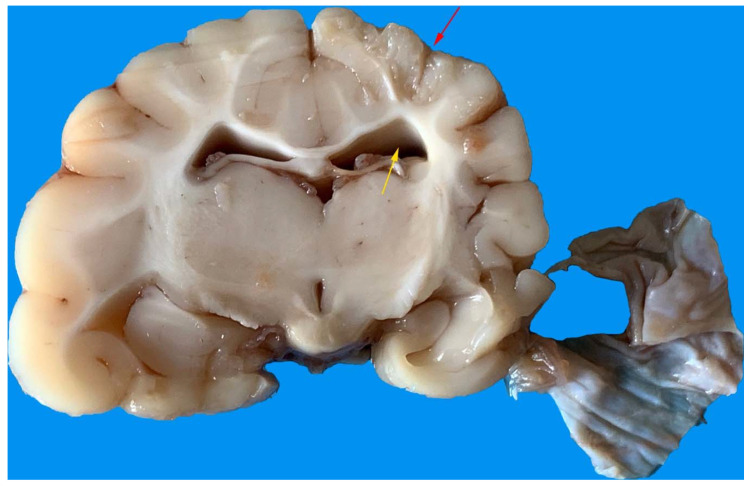
Temporoparietal portion of the left lobe of the brain with less compact areas (red arrow). The ventricles and furrows were enlarged (yellow arrow).

The brain presented macroscopic injuries in all foxes, but one. The lesions were mild and mainly concerned the temporoparietal portion of the left lobe of the brain with distorted cerebral convolutions. Some areas of convolutions appeared depressed or less consistent. The ventricles and furrows on the cutting surface were enlarged due to the presence of a citrine liquid (Figure 5).

**Figure 6:**
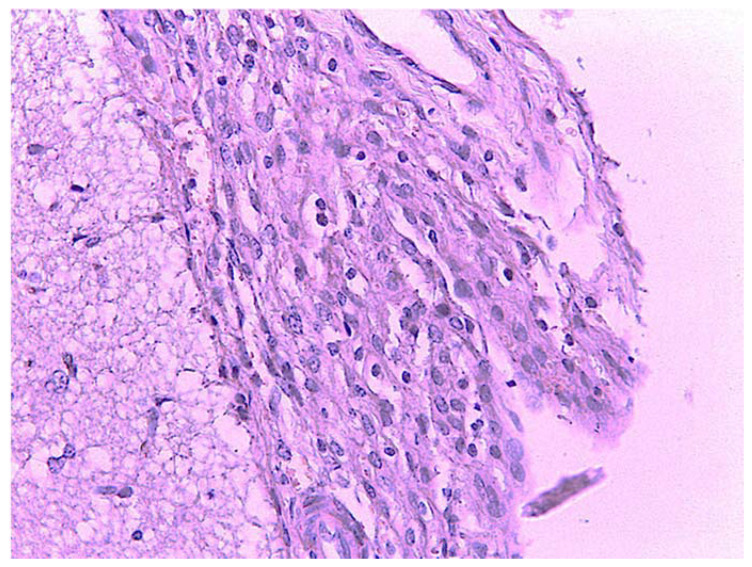
Leptomeninges: macrophage–lymphocytic infiltrate. H.E. Magnification: 40×.

The microscopic examination of the subdural space revealed an increase in the erythrocytes and macrophage–lymphocytes of the leptomeninges (Figure 6). Occasionally, there were scattered hemosiderin-laden macrophages within the brain parenchyma.

**Figure 7:**
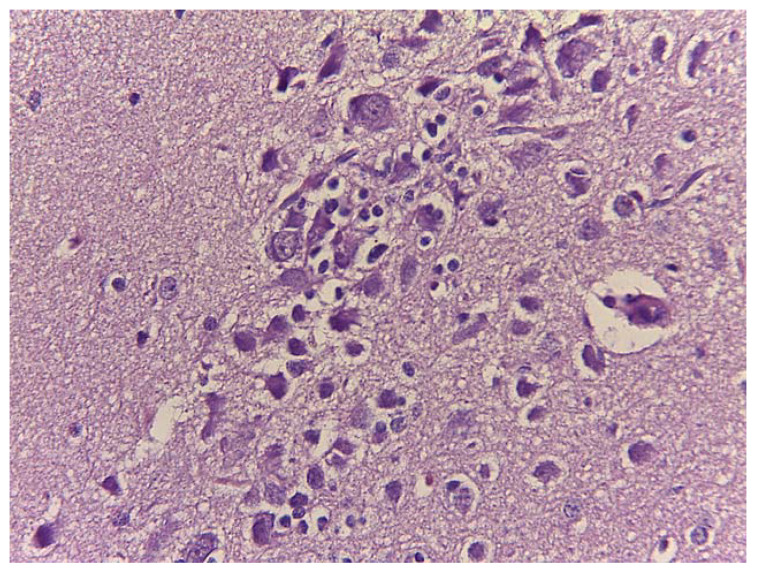
Neurons shrunken and degenerated surrounded by a reactive glia. H&E. Magnification: 40×.

**Figure 8:**
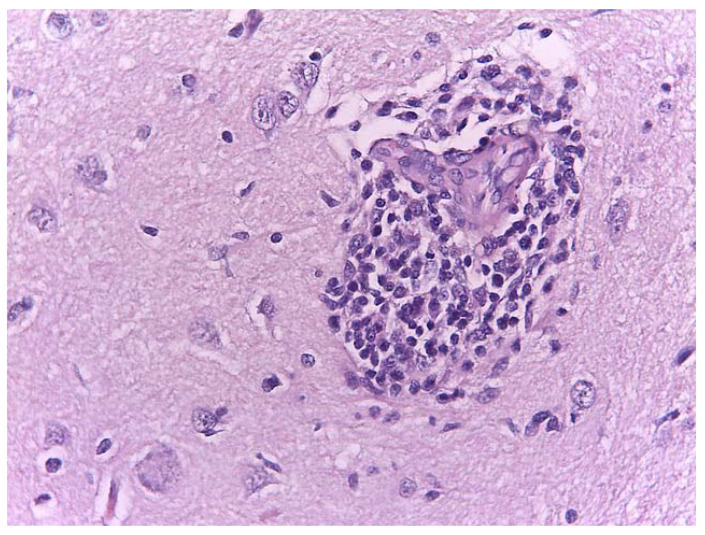
Perivascular cuffing of macrophage–lymphocyte cells. H&E. Magnification: 40×.

A marked loss of normal cortical lamination was observed. The neurons appeared shrunken, coarctated and disorganized with irregular edges. Eosinophilic granules or globules filled the cytoplasm. The nuclei were pyknotic. Neurons, focally distributed, were surrounded by reactive glia and showed elongated and hyperchromatic nuclei. These were typical signs of neuronal degeneration and necrosis (Figure 7). Perivascular thickening cuffs of inflammatory cells (Figure 8) were also observed.

**Figure 9:**
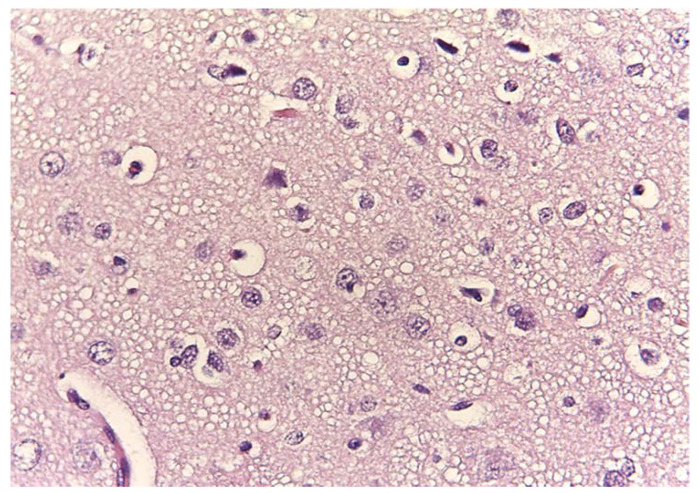
Spongy degeneration. Magnification: 40×.

A spongy degeneration with vacuolization, variable in size and number, was observed, especially in the gray matter (Figure 9).

Cubic ciliated cells and few paving cells were observed in the ependyma lining the lateral ventricles. The white matter astrocytes and cortical areas were rarefied and irregularly arranged. In the cerebellum, the granular layer was characterized by low cellularity without significant alterations of Purkinje cells and molecular layers.

## 4. Discussion

In this study, we retrospectively evaluated the clinical records of red foxes to characterize CT findings in a cohort of foxes with head trauma and identify survival rates and prognostic indicators.

Despite traumatic brain injury frequently occurring in dogs and cats with a high mortality rate [7], mainly due to the secondary lesions that occur within minutes of the injury [7,8], scanty data are available for traumatized foxes.

In our study, the clinical signs observed at admission were mostly semi-comatose and comatose status and, more rarely, seizures, nystagmus and head tilt. In dogs, head trauma symptoms include sudden decrease in mentation, pupillary light reflex, decerebrate posture (opisthotonos with hyperextension of all four limbs and loss of physiologic nystagmus related to increased intracranial pressure [5,9]. The primary injury occurs immediately after trauma due to the physical insult and the disruption of intracranial structures at the time of impact [5]. The most severe forms of primary brain injury are lacerations, but vasogenic edema may also occur if there is a direct vascular injury followed by intracranial hemorrhage [7,9].

Secondary injuries occur in the minutes or even days following trauma and involve the activation of several biomechanical mechanisms that together act to perpetuate brain lesions and whose course is a determining factor for the patient’s prognosis [7,10].

Seizures can occur after head trauma injury in dogs and even in some idiopathic, infectious or congenital conditions [11,12,13,14]. In a fox, a central vestibular syndrome after ischemic infarct of the right caudal artery has been reported [15].

In this study, we used the MGCS scoring system to assess the mental status and provide a prognosis. This scoring system has been previously validated in dogs [16], and it was able to offer reasonable discriminatory performance even in the absence of a specific history or injury pattern consistent with head trauma [17]. The predictive value of the MGCS is restricted to the first 48 h of hospitalization (this is a limitation of the method) [18]. Unfortunately, it was impossible to assess the time elapsed between trauma and clinical evaluation in this cohort of rescued animals since it can take several days between the time of the injury and the rescue. Regardless, the score at any given time may indicate the severity of the underlying brain injury [19].

CT scanning helps identify fractures, parenchymal damages, and hemorrhages (intra-axial and extra-axial) [5,19]. Chai et al. [18] developed a seven-point prognostic scale, including points for hemorrhages, midline shift or lateral ventricle asymmetry, cranial vault fractures, depressed fractures, and infratentorial lesions, but it has not yet been validated.

Multiple brain injuries with hemorrhage and brain edema are responsible for the appearance of secondary autolytic processes that eventually lead to death. Secondary edema and swelling lead to increased intracranial pressure, causing transtentorial and/or transforaminal herniation. These lesions are responsible for the clinical signs of a Cushing’s reflex and changes in mentation and in pupil size [20].

The CT scans performed in our study showed fractures at different healing stages on cranial bones, but as the diagnostic investigations were conducted long after the head trauma, bone repair processes were constantly evident. The time elapsed from trauma to diagnostic imaging investigation also influenced the results of acute bleeding, confirmed histologically by the presence of macrophages containing hemosiderin, which were inversely proportional to the age of the outbreak examined.

CT attenuation is linearly related to hemoglobin concentration that, when greater than 9–11 g/dL, results in a hematoma appearing as a hyperintense area in the brain. This correlates well with the high concentration of hemoglobin noted as red blood cells aggregate in an acute hematoma [21]. Decreased hemoglobin concentration, as hemoglobin is metabolized to methemoglobin, results in isoattenuating (subacute) to hypoattenuating (chronic) hematomas. When the hematomas are small, without mass effect, and isointense to the brain, MRI offers the greatest advantage in detecting lesions [21]. Agreement between CT and MRI was reported to be substantial in identifying the presence of a lesion and in determining whether solitary or multiple lesions are involved [22].

Although midline shift was not associated with survival, this variable is similar to ventricular asymmetry, which indicates a mass effect [18].

Posttraumatic hydrocephalus (PTH) is typically characterized by progressive accumulation of cerebrospinal fluid (CSF) and ventriculomegaly secondary to disorders involving CSF circulation and malabsorption. PTH can disrupt brain function or metabolism, delay clinical improvement, and worsen TBI outcomes if not detected and effectively treated in time [23]. In men, clinical signs and CT findings of hydrocephalus have been reported from 7 days to 30 months after traumatic brain injury [24]. In our study, we performed CT scans on average 260 days after fox rescue, and we speculate that persisting clinical signs could be mostly attributed to this condition. Another hypothesis is that cerebral atrophy could have occurred secondary to brain trauma. Compensatory hydrocephalus occurs when there is primary loss or absence of normal brain parenchyma, and CSF accumulates secondarily in the void. This condition may be acquired and occurs secondary to infarction, trauma, and inflammatory processes [21,25,26]. Posttraumatic hydrocephalus must be differentiated from ventricular enlargement secondary to posttraumatic cerebral atrophy. It has been reported that if the study of CFS dynamics reveals a normal pressure and outflow resistance, a posttraumatic ventriculomegaly secondary to an atrophic process should be considered [27]. It should be a consequence of the severe injury, showing the involvement of one or more pathogenetic factors responsible for hydrocephalus, such as meningitis, traumatic subarachnoid hemorrhage, fourth ventricle obstruction by cerebellar contusion and intracranial hematoma with contralateral ventricular dilatation [24]. Ventricular diameter does not correlate well with clinical signs [28]. In our study, no correlation was found between the severity of hydrocephalus and clinical signs or with survival rate. Conversely, in humans, PTH can lead to unfavorable outcomes [23].

In this study, only two foxes were alive at the time of writing. One of them was released, but one was still captive at OFR because of an inability to autonomously obtain food. Other foxes were euthanized due to the severity of clinical signs.

Hemorrhage and ventricular asymmetry, which both directly impact brain parenchyma, are associated with poor outcomes [18]. Midline shift could be due to edema or tissue loss (posttraumatic porencephaly) [29]. In this study, we observed that chronic foxes with no or mild midline shift had longer survival. However, statistically significant findings should be evaluated in a greater population.

Chai et al. [18] described a scoring system, named the KCTS system, which provides a qualitative outcome assessment for head trauma in dogs based on significant associations between abnormal CT findings and both short- and long-term survival during statistical analysis. Their CT data revealed that hemorrhage (intra-axial or extra-axial) is significantly and negatively associated with short-term survival, whereas ventricular asymmetry is significantly and negatively associated with long-term survival. All other CT abnormalities, such as number and location of hemorrhage, hypodense areas, midline shift, cranial vault and facial bone fracture, are not associated with short- or long-term survival. In a recent study, the KCTS system evaluated the accuracy of the KCTS in making short- and long-term prognoses in dogs with acute traumatic brain injury. The authors found a significant negative association was found between KCTS and both short- and long-term survival, justifying performing head CT and applying the KCTS routinely in prognostication of dogs with acute traumatic brain injury [30]. However, in our study, we evaluated chronic head trauma in a small cohort of animals. Thus, further studies should be performed to confirm this scoring system’s feasibility—even for evaluation of prognosis in chronic trauma.

The low cellularity of the granule cell layer observed at the histological examination may be associated with the vestibulocerebellar signs [14] since cerebellar abnormalities linked with ataxia are frequently described in dogs, including degeneration, hypoplasia, and localized defects [14].

In veterinary diagnostic imaging, X-rays, CT and MRI have been largely employed for the study of brain injury. X-ray views of the head are the first-level imaging technique in patients with head trauma. However, both CT and MRI provide thorough information of the neuroanatomy, giving more advantages over the X-rays because they avoid superimposition of surrounding tissue by obtaining cross-sectional or other plane sections. MRI provides more significant details of soft tissues, but the equipment is still not very widespread, needs a longer execution time, higher costs and general anesthesia is mandatorily required; thus, it may, therefore, not be an appropriate choice for patients in poor condition. Conversely, CT is a more available technique among veterinary clinics, and because it can be performed in dozens of seconds, anesthesia can be avoided in critical patients [5,18]. Furthermore, images obtained from CT studies provide significant findings of the head bone structure, nevertheless providing essential details also for soft tissues. In fact, Gielen et al. (2013) evaluated that CT and MRI may be considered concordant for the most diagnostically important imaging findings in head injury in dogs and cats [22].

This study has some limitations. The small number of foxes included in the present study limited the statistical analysis in detecting differences in the prevalence of CT findings between survivors and non-survivors and some CT abnormalities that appeared more common in non-survivors than in survivors. CT scan was performed on average 260 days after admission to OFR. We speculate that the clinical symptoms at admission and the elapsed time dramatically influenced the acute CT findings, notably influencing the prognostic value of diagnostic imaging.

## 5. Conclusions

CT scanning is essentially helpful and strongly suggested in diagnosing chronic brain lesions and their effects on prognostic judgment for animals released to wildlife environments.

## Figures and Tables

**Table 1:** Mental status at admission.

Mental Status				
Unremarkable	Depressed	Delirium	Stupor or Semi-Comatose	Comatose
1	0	2	2	4

**Table 2:** Computed tomography (CT) findings.

	Mild	Moderate	Severe
Cranial vault fractures (depression)	3 (50%)	1 (17%)	2 (33%)
Abnormalities of the parenchyma			
	Mild	Moderate	Severe
Cerebral hemisphere or cerebellum as a single lesion	3 (50%)	2 (33%)	1 (17%)
Multiple lesions	2 (33%)	3 (50%)	0
Intracranial hemorrhage			
	Mild	Moderate	Severe
Intra-axial	3 (50%)	3 (50%)	0
Extra-axial	2 (33%)	1 (17%)	1 (17%)
Other findings			
Midline shift	2 (33%)	1 (17%)	0
Lateral ventricle asymmetry	2 (33%)	1 (17%)	2 (33%)
Hydrocephalus	1 (33%)	1 (17%)	2 (33%)

1 = mild—slightly visible, 2 = moderate—modestly appreciable, and 3 = severe—clearly visible.

## Data Availability

Data is contained within the article.
